# Transforming Growth Factor-β Activated Kinase 1 (Tak1) Is Activated in Hepatocellular Carcinoma, Mediates Tumor Progression, and Predicts Unfavorable Outcome

**DOI:** 10.3390/cancers14020430

**Published:** 2022-01-15

**Authors:** Dirk Andreas Ridder, Lana Louisa Urbansky, Hagen Roland Witzel, Mario Schindeldecker, Arndt Weinmann, Kristina Berndt, Tiemo Sven Gerber, Bruno Christian Köhler, Federico Nichetti, Annekathrin Ludt, Nadine Gehrke, Jörn Markus Schattenberg, Stefan Heinrich, Wilfried Roth, Beate Katharina Straub

**Affiliations:** 1 Institute of Pathology, University Medical Center of the Johannes Gutenberg University, 55131 Mainz, Germany; lana.urbansky@t-online.de (L.L.U.); hagen.witzel@unimedizin-mainz.de (H.R.W.); Mario.Schindeldecker@unimedizin-mainz.de (M.S.); k.berndt@students.uni-mainz.de (K.B.); tiemo.gerber@unimedizin-mainz.de (T.S.G.); wilfried.roth@unimedizin-mainz.de (W.R.); 2 Tissue Biobank, University Medical Center of the Johannes Gutenberg University, 55131 Mainz, Germany; 3 Department of Internal Medicine, University Medical Center of the Johannes Gutenberg University, 55131 Mainz, Germany; arndt.weinmann@unimedizin-mainz.de (A.W.); nadine.gehrke@unimedizin-mainz.de (N.G.); joern.schattenberg@unimedizin-mainz.de (J.M.S.); 4 Department of Medical Oncology, National Center for Tumor Diseases, University Hospital Heidelberg, 69120 Heidelberg, Germany; bruno.koehler@nct-heidelberg.de; 5 Medical Oncology Department, Fondazione IRCCS Istituto Nazionale dei Tumori di Milano, 20133 Milan, Italy; federico.nichetti@dkfz-heidelberg.de; 6 Computational Oncology, Molecular Diagnostics Program, National Center for Tumor Diseases (NCT) and German Cancer Research Center (DKFZ), Im Neuenheimer Feld 280, 69120 Heidelberg, Germany; 7 Institute of Medical Biostatistics, Epidemiology, and Informatics (IMBEI), University Medical Center Mainz, 55131 Mainz, Germany; anneludt@uni-mainz.de; 8 Department of General, Visceral and Transplant Surgery, University Medical Center of the Johannes Gutenberg University, 55131 Mainz, Germany; stefan.heinrich@unimedizin-mainz.de

**Keywords:** Tak1, MAP3K7, Cyld, hepatocellular carcinoma, HCC, prognosis, biomarker, VETC

## Abstract

**Simple Summary:**

Chronic inflammation is known to drive cancer initiation and progression in the liver and other organs. In different genetic mouse models, the role of the pro-inflammatory kinase Tak1 in liver cancer development has been controversial so far. To clarify the role of Tak1 in human hepatocellular carcinoma (HCC), we investigated the expression of Tak1 in a large and clinicopathologically well-characterized patient cohort with HCC. In human livers and HCCs, Tak1 is predominantly present in its isoform Tak1A localizing to the cell nucleus. Tak1 is upregulated in HCCs of the diethylnitrosamine mouse model as well as in human HCCs, independent of etiology, and is further induced in distant metastases. Overexpression of the isoform Tak1A in the HCC cell line Huh7 resulted in increased tumor cell migration, whereas overexpression of full-length Tak1 had no significant effect. In human HCCs, high nuclear Tak1 expression is associated with vascular invasion and short overall survival.

**Abstract:**

Although knowledge on inflammatory signaling pathways driving cancer initiation and progression has been increasing, molecular mechanisms in hepatocarcinogenesis are still far from being completely understood. Hepatocyte-specific deletion of the MAPKKK Tak1 in mice recapitulates important steps of hepatocellular carcinoma (HCC) development, including the occurrence of cell death, steatohepatitis, dysplastic nodules, and HCCs. However, overactivation of Tak1 in mice upon deletion of its deubiquitinase Cyld also results in steatohepatitis and HCC development. To investigate Tak1 and Cyld in human HCCs, we created a tissue microarray to analyze their expression by immunohistochemistry in a large and well-characterized cohort of 871 HCCs of 561 patients. In the human liver and HCC, Tak1 is predominantly present as its isoform Tak1A and predominantly localizes to cell nuclei. Tak1 is upregulated in diethylnitrosamine-induced mouse HCCs as well as in human HCCs independent of etiology and is further induced in distant metastases. A high nuclear Tak1 expression is associated with short survival and vascular invasion. When we overexpressed Tak1A in Huh7 cells, we observed increased tumor cell migration, whereas overexpression of full-length Tak1 had no significant effect. A combined score of low Cyld and high Tak1 expression was an independent prognostic marker in a multivariate Cox regression model.

## 1. Introduction

Primary liver cancer constitutes a major global health problem and caused about 782,000 cancer-related deaths in 2018. Hepatocellular carcinoma (HCC) accounts for about 90% of primary liver cancers [1]. Early-stage tumors may be treated by surgical resection and liver transplantation. Furthermore, a considerable proportion of HCC patients benefits from local ablation and transarterial chemoembolization [2]. However, HCC is often diagnosed in an advanced stage and currently available treatment options, such as multi-tyrosine kinase inhibitors (TKIs) and immune checkpoint inhibitors, show only limited clinical efficacy. Especially for TKIs, the early occurrence of drug resistance is a major obstacle. Preclinical models suggest that a combination of TKIs with other targeted molecular therapies may overcome acquired TKI resistance [3].

Although knowledge on signaling pathways driving cancer initiation and progression has been increasing, the molecular mechanisms in hepatocarcinogenesis are still far from being completely understood. In order to unravel the functional role of specific molecular processes, several mouse models for HCC have been created. One genetic model, that has been widely used and analyzed in detail, is the hepatocyte-specific Tak1 knockout mouse [4].

Tak1 is a mitogen-activated protein kinase kinase kinase (MAPKKK) that is activated by pro-inflammatory signaling molecules, such as interleukin 1, tumor necrosis factor, toll-like receptors 2 and 4, and lipopolysaccharides. In turn, Tak1 via mitogen-activated protein kinase kinases (MKK) 4, 6, and 7 activate c-Jun N-terminal kinase (JNK) and p38 mitogen-activated protein kinase (MAPK), and the IkB kinase (IKK) complex, leading to the activation of the proinflammatory transcription factors activator protein-1 (AP1) and NF-kappaB (nuclear factor kappa-light-chain-enhancer of activated B cells) [5]. Furthermore, Tak1 has been demonstrated to elicit pro-survival signals and to inhibit apoptosis and necroptosis [6]. In a variety of tumor types, Tak1 has been identified as a factor associated with unfavorable outcomes and has been implied in tumor metastasis [5,7,8,9]. Upon deletion of Tak1 in hepatocytes, mice develop steatohepatitis, fibrosis, dysplastic nodules, and HCCs [4,10,11]. Comparative studies have shown considerable similarity between HCCs in Tak1-deficient mice and human HCCs [12,13]. Intriguingly, there are studies in mice that showed that also overactivation of Tak1, as a major pro-inflammatory kinase, may lead to steatohepatitis and the development of HCCs [13,14].

Despite detailed molecular analysis of the pathways involved in cancer development in these murine models, Tak1 protein expression in human HCCs has not been thoroughly investigated in a large and clinicopathologically well-characterized cohort of patients. Therefore, we created a large tissue microarray comprising over 850 HCC from 561 patients, including primary and recurrent HCCs, tumor thrombi, lymph node and distant metastases, as well as surrounding non-neoplastic liver tissue, and correlated Tak1 expression with comprehensive clinical and histopathological parameters. In situ Tak1-expression in human HCC was compared with a diethylnitrosamine (DEN)-based HCC mouse model treated with a high-fat diet and normal chow diet. To analyze the functional role of Tak1 in vitro, we overexpressed full-length Tak1 (Tak1B) and its isoform Tak1A in Huh7 cells and analyzed cell proliferation and motility.

## 2. Materials and Methods

### 2.1. Patients and Samples

Tissue samples from 561 HCC patients who underwent tumor resection at the University Medical Center Mainz from 1997 to 2017 were provided by and in accordance with the regulations of the Tissue Biobank of the University Medical Center Mainz after approval by the local ethics committee (Ethik-Kommission der Landesärztekammer Rheinland-Pfalz, 837.146.17 (10980), as well as addendum 2018-13857_1 to DAR and BKS). Clinical data of HCC patients, including survival, were retrieved from a prospectively populated clinical database [15]. Patient records and information were anonymized and de-identified prior to analysis. The mean duration of follow-up was 55.2 months.

### 2.2. Immunohistochemistry

A tissue microarray (TMA) was created, comprising at least two cores of the primary tumor and surrounding non-neoplastic liver tissue of each patient, as well as of relapse tumors, lymph node and distant metastases, and larger tumor thrombi, if available [16]. Antigen retrieval was performed using citrate buffer, pH = 6.1 (Dako, Santa Clara, CA, USA, #GV805) or Tris/EDTA buffer, pH 9 (Dako, #8024), or cell conditioning solution 1 (Roche, Mannheim, Germany, #950-124). After antigen retrieval, tissue microarray slides were incubated with the respective primary antibodies (see Table S1). Staining was performed with an automated staining system (DAKO Autostainer Plus, Agilent Technologies, Santa Clara, CA, USA) and the Dako EnVision FLEX staining system (Agilent Technologies) in accordance with the manufacturers’ instructions. TMA slides were digitalized using the NanoZoomer-Series Digital slide scanner (Hamamatsu Photonics, Hamamatsu, Japan) prior to image analysis. Immunoreactivity was either rated semi-quantitatively according to Remmele et al. [17] or in the case of Cyld, Ki67, and CD34, digital image analysis was performed using the HALO platform (Indica Labs, Corrales, NM, USA), including the TMA module and the CytoNuclear v1.6 module. Missing or erroneous cores, for example, with extensive necrosis, were excluded from the analysis. In the case of Cyld, the average cytoplasmic and nuclear densities were determined; in the case of Tak1 and Ki67, positive cell nuclei were counted; in the case of CD34, the stained area was quantified.

### 2.3. Immunoblotting

For the isolation of proteins from cryopreserved human liver and HCC tissue, 100 cryosections (5 μm) were prepared with a cryostat (Leica CM3050 S, Wetzlar, Germany) and homogenized with a homogenizer (Precellys 24, Bertin Instruments/VWR, Darmstadt, Germany) at 6500 rpm for 20 sec in a lysis buffer (50 mM Tris, 100 mM NaCl, 15 mM EGTA, 1% Triton X-100, pH = 8). Cultured cells were homogenized with a digital sonifier (Branson SLPe, Fisher Scientific, Waltham, MA) for 10 sec at maximum power in lysis buffer. After centrifugation, the supernatant was incubated with hot 2× Laemmli buffer for 5 min at 95 °C, and then loaded on SDS-PAGE gels. Proteins were transferred to nitrocellulose membranes, which were blocked with 5% dry-milk, incubated with either an anti-Tak1 (Novus Biologicals/Bio-Techne, Wiesbaden, Germany, #JM73-19, 1:1000 dilution) or an anti-Actin-antibody (Sigma-Aldrich, Taufkirchen, Germany, #MAB1501; 1:10,000 dilution) for one hour at room temperature, and subsequently with HRP-conjugated secondary antibodies for 1–2 h at room temperature. For detection, enhanced chemiluminescence and a digital detection system (Fusion Solo S, Vilber, Eberhardzell, Germany) were used.

### 2.4. Real-Time RT-PCR

RNA was isolated from cryopreserved human tissue by performing 50 cryosections (5 µm) with a cryostat (Leica CM3050 S, Wetzlar, Germany), homogenization in 1 mL TRIzol Reagent (Invitrogen, Carlsbad, CA, USA), and RNA isolation according to the manufacturer’s instructions. Two micrograms of RNA per sample were transcribed with the High-Capacity cDNA Reverse Transcription Kit (Applied Biosystems, Waltham, MA, USA), according to the manufacturer’s instructions. The following primers were used for quantitative RT-PCR [18]: TAK1-Exon-12 forward: 5′-CCTATTCCAAGCCTAAACGG-3′, reverse: 5′-GATATGACGATCTCAGGGACA-3′; and TAK1-Exon-2 forward: 5′-TGTTGGAAGAGGAGCCTTTG-3′, reverse: 5′-ACGCTTTCCTCTCAGATTCAC-3′ as well as RNA18S forward: 5′-CATGGCCGTTCTTAGTTGGT-3′ and RNA18R reverse: 5′-ATGCCAGAGTCTCGTTCGTT-3′.

### 2.5. Mouse Experiments

All animals were held and bred according to the criteria outlined by the “Guide for the Care and Use of Laboratory Animals”. Studies were approved by the committee for experimental animal research (Landesuntersuchungsamt Rheinland-Pfalz, G18-1-066; as well as Regierungspräsidium Karlsruhe, G-11/14). Diethylnitrosamine (DEN, 25 mg/kg body weight dissolved in PBS) was given intraperitoneally (i.p.) to 2-week-old male C57BL6/J mice. From 6 weeks of age, the mice were fed either a high-fat diet (HFD; 35.5% w/w crude fat (58 kJ%), metabolizable energy (ME): 5.45 kcal/g), and fructose/glucose (55/45% *w*/*v*) enriched drinking water or a corresponding control diet (CD; 5.4% *w*/*w* crude fat (13 kJ%), ME: 3.74 kcal/g) and plain water until the mice were sacrificed at 30 weeks of age. The composition and energy density of the diets (ssniff Spezialdiäten GmbH, Soest, Germany) are listed in Supplementary Table S2. For the duration of the study, all mice were kept on a 12-h light/dark cycle with constant temperature (22 ± 2 °C) and humidity (55 ± 10%) and with free access to the experimental diets and water. Biometric data including body weight and food consumption was measured weekly. All mice fasted overnight before sacrifice for collection of blood and liver samples. For histology, liver tissue specimens from the left lateral lobe were investigated. Steatosis was scored as follows: 0 = no steatosis; 1 = 1–10%; 2 = 11–50%; 3 = more than 50% of the hepatocytes with neutral fat deposition. Inflammation was scored according to the following criteria: 0 = no inflammation; 1 = little portal inflammation, 3–5 single necrotic cells/15 high power fields (HPF), no grouped necrotic cells; 2 = intermediate portal inflammation, 6–9 single necrotic cells/15 HPF and/or one focus of grouped necrotic cells; 3 = severe portal inflammation, ≥ 10 single necrotic cells/15 HPF, more than one focus of grouped necrotic cells. For the use of this study, a score reflecting steatohepatitis was calculated as the sum of the scores of steatosis and inflammation. DEN-treated mice developed the whole spectrum of pre-neoplastic and neoplastic lesions from dysplastic foci and nodules to early and advanced HCCs. For the determination of tumor size and area as well as intratumoral Tak1 immunoreactivity, the whole spectrum of lesions was considered.

In a second experimental group, a single dose of DEN (25 mg/kg body weight dissolved in PBS) was applied by i.p. injection at day 7 postpartum. Additionally, phenobarbital (0.05% *w*/*v*) was continuously added to the drinking water from the age of 3 weeks. Mice were sacrificed at the age of 6 months.

Cyld-liver parenchymal cell-deficient (Cyld^ΔLPC^) mice were generated as described previously [19]. AlbCre transgenic mice were crossed to mice carrying loxP sites flanking exon 7 of Cyld, leading to a liver parenchymal cell-specific alternative splicing from exon 6 to exon 9 and knockout of full-length Cyld [20,21]. Mice were sacrificed at the age of 6 months.

### 2.6. Plasmids

The isoforms Tak1A and Tak1B were amplified from cDNA of HEK293T cells with the following primers:

TAK1 forward: 5′-CATGGATCCGCCACCATGTCTACAGCCTCTGCCGC-3′
TAK1 reverse: 5′-CATCTCGAGTGAAGTGCCTTGTCGTTTCT-3′
TAK1STP reverse: 5′-CATCTCGAGTCATGAAGTGCCTTGTCGTTTC-3′

Via the restriction enzymes BamHI and XhoI (New England Biolabs, Frankfurt a. M., Germany), the amplicons were subcloned into the pcDNA3-Flag vector.

### 2.7. Cell Culture

Cultured cells of the lines Huh7 and HepG2 were cultivated in DMEM (Gibco), supplemented with 10% fetal bovine serum (Sigma-Aldrich, Taufkirchen, Germany) and 1% of 10,000 U/mL penicillin and 10 mg/mL streptomycin (Sigma-Aldrich, Taufkirchen, Germany, #P0781), at 5% CO_2_ and 37 °C. Huh7 cells were chosen for migration and proliferation experiments due to higher transfection rates. Cell lines were tested for mycoplasma contamination on a regular basis. Transfection was carried out with polyethyleneimine.

### 2.8. Migration Assay

Two million, six hundred thousand Huh7 cells were seeded in 10 cm plates and transfected with 10 µg of the respective plasmids with polyethyleneimine (Sigma-Aldrich, #408727) as specified by the manufacturer. In brief, 1 µg DNA was diluted in 18 µL PBS (solution A) and further mixed with solution B (2 mM PEI (Sigma-Aldrich, 40872-7) in PBS) by pipetting up and down and additional vortexing for 5 s. The mixture was incubated for 5 min at RT and pipetted dropwise onto the cells. Twenty-four hours later, cells were trypsinized, counted, and seeded in a culture-insert (ibidi culture-insert 2 well, ibidi GmbH, Martinsried, Germany) at a density of 35,000 cells per compartment of the insert. Another 24 h later, inserts were removed, and images were taken at 0, 14, and 22.5 h with a Nikon Eclipse TS100 microscope with a 10×/0.25 objective equipped with a camera (Progres Gryphax Subra, Jenoptik, Jena, Germany) and the software Progres Gryphax, Version 2.0.0.68, Jenoptik, Jena, Germany. The average migration distance after 14 h was calculated from the mean gap width that was determined with the software ImageJ [22]. Data from three independent experiments were combined.

### 2.9. Immunofluorescence Microscopy

Huh7 and HepG2 cells were seeded on 13 mm coverslips in 12-well plates. Forty-eight hours after transfection with polyethyleneimine, according to the manufacturer’s instructions (as described above), cells were washed with PBS, fixed for 10 min with 3.7% formaldehyde in PBS, subsequently washed with PBS, permeabilized, and blocked with 10% FBS, 0.1% Triton X-100 in PBS at 37 °C. After incubation with the primary antibodies mouse anti-Flag (Sigma-Aldrich, #F1804, 1:100) and rabbit anti-phospho-histone H3 (Ser10) (Sigma-Aldrich, #F1804, 1:250) in blocking solution at RT for 1 h and a brief wash with 0.1% Triton X-100 in PBS, cells were incubated with the Alexa Fluor^®^488 goat anti-mouse and Alexa Fluor^®^546 goat anti-rabbit secondary antibodies (1:250, Invitrogen, Carlsbad, CA, USA) and the nuclear dye DAPI in 1% FBS, 0.1% Triton X-100 in PBS at RT for 1 h. After an additional washing step with 0.1% Triton X-100 in PBS, cells were mounted with MOWIOL, and images were obtained with a BZ-9000E microscope (Keyence, Osaka, Japan).

### 2.10. Estimation of Protein Activity in the Cancer Genome Atlas (TCGA) Cohort

Molecular and clinical data of the hepatocellular carcinoma cohort (LIHC) of the TCGA project were retrieved via the Bioconductor curated TCGA data package [23,24]. RNA sequencing data were available for 371 individual tumor samples. Upper quartile normalized RSEM TPM gene expression data were used for comparative analysis of protein activity and mRNA expression. The activity of 5424 proteins was inferred from RNA sequencing data by Master Regulator Analysis utilizing the VIPER algorithm, as described previously [25,26].

### 2.11. Statistical Analysis

All statistical analyses concerning the human patient cohort were performed within the R environment for statistical computing (version 4.1.1, R Foundation for Statistical Computing, Vienna, Austria) [27]. Mouse and cell culture data were analyzed with GraphPad Prism (GraphPad Software Inc., San Diego, CA, USA). The non-parametric Mann–Whitney U test was applied to compare differences between two independent groups when dependent variables were either ordinal or continuous. The non-parametric Wilcoxon signed-rank test was used to determine whether two dependent samples were selected from populations having the same distribution. The Kruskal–Wallis test was applied to compare two independent groups, which consisted of one dependent scale variable and one explanatory nominal variable with 3 or more levels. Benjamini–Hochberg corrections were applied to reduce the effects of multiple testing and control for the false discovery rate. Categorical variables were compared using the χ^2^ test or Fisher’s exact test. *p*-values ≤ 0.05 were considered statistically significant. Tak1 and Cyld protein expression was dichotomized utilizing the Charité Cutoff Finder functions to provide a significant distinction between the high and low expression levels based on survival outcome [28]. Overall survival was calculated as the interval between initial diagnosis and death, regardless of etiology or the last follow-up. The Kaplan–Meyer method was used to analyze overall survival, and differences were evaluated by the log-rank test. Uni- and multivariate Cox regression analysis was conducted for 561 patients with a mean survival time of 55.2 months using the functions coxph from the R package survival (version 3.2.13, R Foundation for Statistical Computing, Vienna, Austria). The proportional hazards assumption was assessed with scaled Schoenfeld residuals. Concerning the covariates VETC pattern, ECOG group, and NASH, we found highly significant relationships between residuals and time, as evidence for the violation of the proportional hazards assumption. These factors were, therefore, excluded from the Cox regression analysis. For AFP serum levels, we observed a *p*-value of 0.03. Because of its high clinical relevance and low significance level, we retained this factor in our model. In order to create a significant multivariate Cox model, a stepwise variable selection was performed by employing the stepwise backward model selection by the Akaike information criterion (AIC) method from the R package MASS (version 7.3.54, R Foundation for Statistical Computing, Vienna, Austria) [29]. At last, the immunohistochemically determined markers were added to the statistical model.

## 3. Results

### 3.1. Tak1 Is Induced in Hepatocellular Carcinoma and Further Increased in Distant Metastases

**Figure 1:**
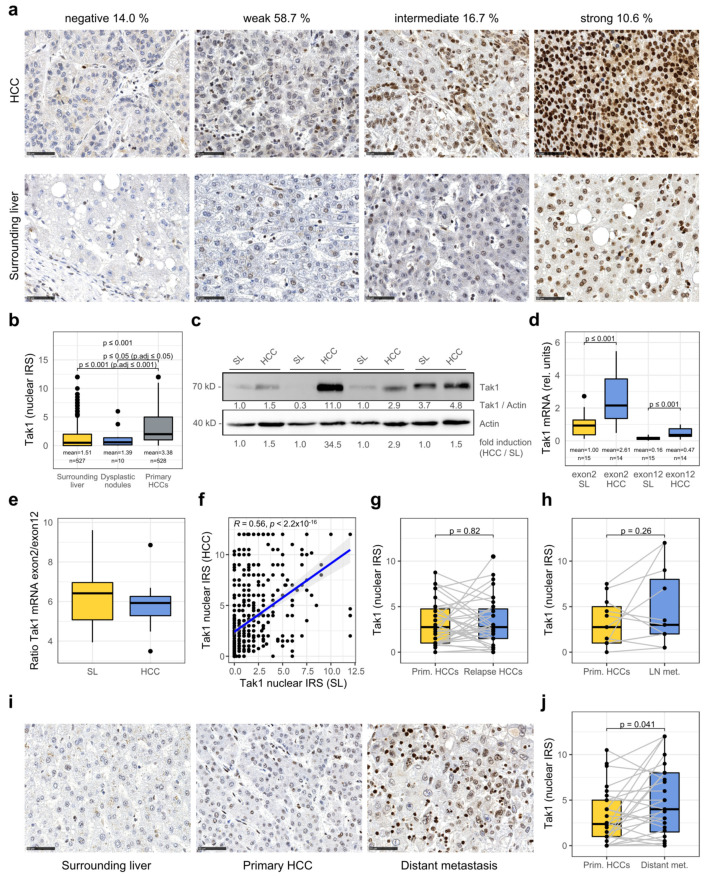
Tak1, especially its isoform Tak1A, is induced in HCC and further increased in distant metastases. (**a**) Representative images of immunohistochemical staining of primary HCCs with different expression levels of Tak1. The percentage of tumors with the indicated expression level is shown. Scale bar: 50 µm. (**b**) Quantitative analysis of nuclear Tak1 expression (immunoreactive score, IRS) in primary HCC compared to non-neoplastic surrounding liver and dysplastic nodules. (**c**) Immunoblot analysis of Tak1 in whole-cell lysates from HCC and surrounding liver (SL) from four individual patients. Actin was used as a loading control. (**d**) Real-time RT-PCR from extracts isolated from HCC and surrounding liver with primers specific for the constant exon 2 and the variable exon 12 of Tak1, indicating a predominance of the isoform Tak1A lacking exon 12 and confirming induction of Tak1 in HCC tissue. (**e**) Ratio of Tak1 mRNA of Exon 2 to Exon 12 in HCC and surrounding liver (real-time RT-PCR) (SL: *n* = 15, HCC: *n*= 14). (**f**) Scatter plot of nuclear Tak1 expression in surrounding liver (x-axis) and HCC (y-axis) (*n* = 501). (**g**) Quantification of Tak1 expression in recurrent HCC compared to primary HCC (*n* = 35). (**h**) Quantification of Tak1 expression in lymph node metastasis compared to primary HCC (*n* = 11). (**i**) Tak1 immunohistochemistry of surrounding liver, HCC, and distant metastasis of one individual patient. (**j**) Quantification of Tak1 expression in distant metastasis compared to primary HCC (*n* = 28).

In order to resolve the apparently contradictory studies in mice with deletion and overactivation of Tak1 both leading to steatohepatitis and the development of HCC [4,10,13,14], we started to investigate the in situ expression of Tak1 in HCC by immunohistochemistry in a large cohort of patients. Therefore, we analyzed a tissue microarray with 871 HCCs of 561 patients with comprehensive clinical and pathological data (for an overview of the patient collective see Ridder et al. [16]). For semiquantitative analysis, we employed the immunoreactive score (IRS) [17]. Fourteen percent of the HCCs presented no detectable Tak1 expression (IRS = 0), whereas 58.7% showed weak (IRS = 1–4), 16.7% intermediate (IRS = 5–8), and 10.6% strong (IRS = 9–12) Tak1 staining, which was predominantly detected in the cell nucleus (Figure 1a). Tak1 immunoreactivity was significantly induced in primary HCCs when compared to the surrounding liver tissue and dysplastic nodules (Figure 1b). We additionally detected increased Tak1 protein levels in HCCs by immunoblotting at a molecular weight of around 70 kDa (Figure 1c, Figure S4).

Different Tak1 isoforms with distinct molecular functions have been described. Tak1A, a splice variant lacking exon 12, has been described to be constitutionally active and to support transforming growth factor β (TGFβ)-mediated NFkB-signaling and epithelial–mesenchymal transition (EMT), whereas the full-length protein (Tak1B) promotes TGFβ-mediated apoptosis [18]. However, to our knowledge, isoform composition in the human normal liver as well as in HCC tissue has not been specifically investigated so far. As we detected only one band by immunoblotting, we employed real-time RT-PCR with primers flanking the variable exon 12 and the constant exon 2 to identify which isoform is in fact present in liver and HCC tissue. By this approach, we confirmed that Tak1 mRNA was significantly induced in HCC compared to the surrounding non-neoplastic liver tissue (Figure 1d) and that the constant exon 2 was much more abundant than the variable exon 12 (Figure 1d,e). We concluded that primarily the constitutively active isoform Tak1A is present in both HCC and liver tissue. As the exclusion of exon 12 has recently been described as an actively regulated biological process, it is also important to note that the ratio between both isoforms is obviously not altered in HCC compared to surrounding liver tissue [18]. Interestingly, Tak1 protein expression in HCC moderately correlated with its expression in the surrounding non-neoplastic liver tissue (Figure 1a,f). We did not detect significantly different Tak1 protein levels in recurrent tumors or lymph node metastases in our HCC collective in situ but found significantly higher Tak1 immunoreactivity in distant metastases when compared to the respective primary HCCs (Figure 1g–j), pointing to a role of Tak1 in tumor progression.

### 3.2. Tak1 Is Induced in a Chemotoxic HCC Mouse Model and Is Further Induced by a High-Fat Diet

**Figure 2:**
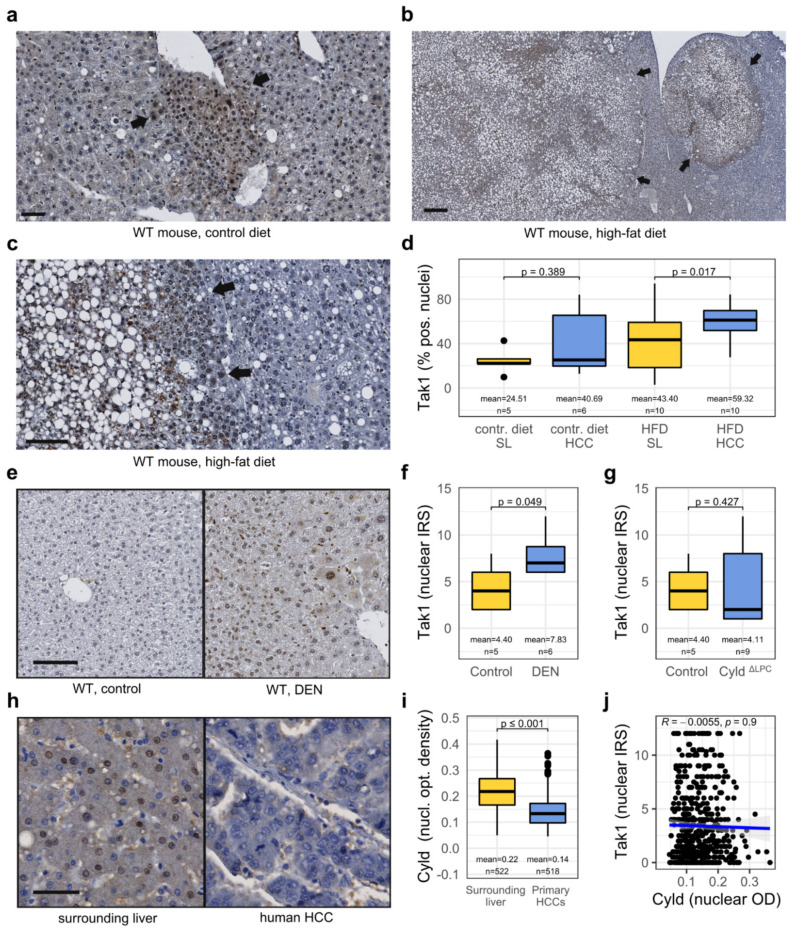
Tak1 is activated in a chemotoxic mouse model of HCC and is further induced upon high-fat, high-carbohydrate (HFD) feeding. (**a**) Tak1 immunohistochemistry of a DEN-induced mouse HCC with vascular invasion (arrows →). A control diet was fed. Scale bar: 50 µm. (**b**) Tak1 immunohistochemistry of two DEN-induced HCCs (arrows) in the liver of a mouse on a high-fat diet. Scale bar: 330 µm. (**c**) Tak1 immunohistochemistry of a DEN-induced HCC (arrows) in the liver of a mouse on a HFD. Scale bar: 100 µm. (**d**) Quantification of Tak1-positive nuclei in the liver of mice treated with DEN in combination with a control diet or HFD. (**e**) Tak1 immunostaining of the liver of a DEN + Phenobarbital treated mouse and a non-treated control. Scale bar: 250 µm. (**f**) Quantification of nuclear Tak1 staining in the liver of DEN + Phenobarbital-treated and non-treated control mice. (**g**) Quantification of nuclear Tak1 staining in Cyld^ΔLPC^ and Cyld^Fl/Fl^ control mice. (**h**) Cyld immunostaining of a human HCC and the surrounding liver tissue. Scale bar: 100 µm. (**i**) Quantification of nuclear Cyld staining in human HCCs and surrounding liver tissue. (**j**) Scatter plot of nuclear Cyld staining (x-axis) and nuclear Tak1 immunoreactivity (y-axis) in human HCCs (*n* = 515).

Several studies have implied Tak1 signaling in chronic liver diseases, such as non-alcoholic steatohepatitis (NASH) [13,14], but in the context of HCC development on the basis of a NASH, it has not been investigated so far. We subjected mice to diethylnitrosamine (DEN)-induced hepatocarcinogenesis and additionally fed either a high-fat, high-carbohydrate diet (HFD), or control diet for 24 weeks resulting in increased fat deposition and inflammatory foci in the liver and larger and more numerous tumors (Figure S1a–c). We detected predominantly nuclear Tak1 expression in the livers and HCCs with the same antibody we used for the previous experiments and observed a trend towards higher Tak1 expression in HCCs compared to the surrounding liver tissue (Figure 2a–d). Paired analysis revealed that Tak1 was significantly induced in HCCs compared to non-neoplastic hepatocytes, at least in the group with high dietary fat and carbohydrate intake (Figure 2d). Additionally, HFD also seemed to result in an increased nuclear Tak1 expression, although the analysis did not reach significance (Figure 2d). Interestingly, nuclear Tak1 expression in the surrounding liver tissue moderately correlated with expression in the respective HCCs (Figure S1d), paralleling the results from human tissue (Figure 1f). As Tak1 has also been implied in the DNA damage response [30], we aimed to investigate the effect of DEN on Tak1 expression in hepatocytes itself. Therefore, we subjected another cohort of mice to DEN treatment and sacrificed them and used an untreated control group at the age of 6 months. Interestingly, we detected a significant increase in Tak1 expression in liver tissue upon DEN treatment (Figure 2e,f).

Recently, several publications have described an important role of the deubiquitinase Cyld in the development of NASH and that Tak1 is mediating its downstream effects [13,14,31]. Cyld removes Lys63-linked polyubiquitin chains of Tak1, which inhibits its auto-phosphorylation and blocks its downstream activation. However, the subcellular localization of Tak1 has not been paid attention to yet. We made use of mice with a liver parenchymal-specific targeted deletion of Cyld, which have been shown to have high Tak1 activity in the liver [19,31]. Deletion of Cyld, however, had no influence on the nuclear localization of Tak1 in hepatocytes (Figure 2g). Additionally, expression of Cyld has been shown to be downregulated in human HCC and low nuclear expression to correlate with worse prognosis [32]. We confirmed the downregulation of nuclear Cyld in human HCC tissue in our patient cohort (Figure 2h,i) and the association of low nuclear expression with reduced overall survival (Figure S1e). However, nuclear expression of Tak1 and nuclear (as well as cytoplasmic) expression of Cyld neither correlated in HCC nor in the adjacent liver tissue (Figure 2j and Figure S1f; not shown). Nuclear translocation of Tak1 may therefore represent an additional regulatory mechanism of Tak1 activity and function that may depend on signaling mechanisms other than deubiquitination by Cyld.

### 3.3. Tak1, Especially Its Isoform Tak1A, Mediates Increased Tumor Cell Migration

**Figure 3:**
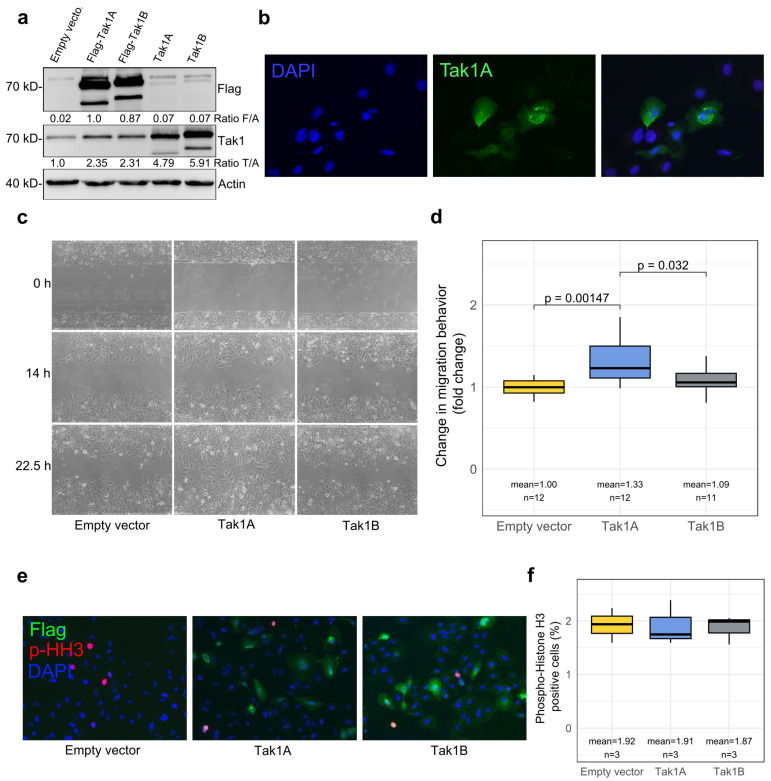
Tak1A mediates increased tumor cell migration. (**a**) Overexpression of the Tak1 isoforms Tak1A and Tak1B in Huh7 cells, with or without Flag-tag, detected by immunoblot with an anti-Tak1 and an anti-Flag antibody. Actin serves as a loading control. The C-terminal Flag-tag hampered the detection of the tagged constructs by the Tak1 antibody directed against the C-terminus of Tak1. Densitometric band intensities in relation to Actin are shown below the respective blot. (**b**) Immunofluorescent staining for Flag of Huh7 cells overexpressing Flag-tagged Tak1A. Cell nuclei were stained by DAPI. (**c**) Representative photographs of a migration assay of Huh7 cells transfected with Tak1A or Tak1B or the empty vector. Gap closure was monitored at the indicated points of time. (**d**) Quantification of mean migration distance of Huh7 cells transfected with Tak1A or Tak1B or the empty vector. Fold changes in relation to the empty vector control are depicted. (**e**) Immunofluorescent staining for Flag and the proliferation marker phospho-histone H3 of Huh7 cells overexpressing Flag-tagged Tak1A or Tak1B or empty vector-transfected controls. Cell nuclei were stained by DAPI. (**f**) Quantification of phospho-histone H3 positive Huh7 cells overexpressing Tak1A or Tak1B or empty vector transfected.

To further investigate the function of both Tak1 isoforms in HCC cells, we overexpressed both Tak1A and Tak1B in Huh7 cells, either with or without a C-terminal Flag-tag. In immunoblot analysis, the same anti-Tak1 antibody detected the non-tagged isoforms at the expected molecular weight of about 70 and 72 kDa, thereby also proving antibody specificity (Figure 3a). The C-terminal Flag-tag, however, hampered detection with the anti-Tak1 antibody directed against an epitope close to the C-terminus, whereas an anti-Flag antibody also revealed the presence of the overexpressed tagged constructs (Figure 3a). Similar to human liver and human HCC tissue, in mock-transfected Huh7 cells, we detected only one band corresponding to the prevalence of the Tak1A isoform, which was confirmed by RT-PCR with exon 2- and exon 12-specific primers as described above (Figure S2e). Unlike the immunohistochemistry in human liver in situ, immunofluorescence microscopy in cultured cells revealed a predominantly cytoplasmic localization of both Tak1 isoforms (Figure 3b).

Overexpression of the Tak1A isoform resulted in significantly increased migratory activity. By contrast, upon overexpression of the full-length isoform Tak1B, migratory activity did not significantly differ from the mock-transfected control group (Figure 3c,d, Figure S4). The proliferation rate, as analyzed by phospho-histone H3 staining, did not show significant differences upon overexpression of either Tak1A or Tak1B, respectively (Figure 3e,f). Increased migratory behavior upon the upregulation of Tak1A may therefore constitute one mechanism for the malignancy of HCC cells and may be involved in tumor progression (Figure 1i,j).

### 3.4. High Tak1 Expression Is Associated with Unfavorable Prognostic Factors and Reduced Overall Survival

Tak1 expression in human tissue was then correlated with clinicopathologic parameters. Tak1 was significantly induced in human HCCs in situ regardless of their etiology (Figure S1h–l). However, HCCs with high nuclear Tak1 expression occurred more often in older patients and less often in association with hepatitis C (HCV) infection (see Table 1). In line with this finding, nuclear Tak1 levels demonstrated a weak correlation with age, both in HCC (Figure S1g) and in surrounding liver tissue (not shown) and were significantly lower in patients with chronic hepatitis C, both in respective HCCs and in surrounding non-neoplastic liver tissue (Figure S2a,b). No significant association was detected with other etiological or liver-related serum parameters (such as alcohol abuse, hepatitis B, hemochromatosis, NASH, alpha-fetoprotein (AFP) serum concentrations, liver enzymes) (Table 1 and Figure S2c,d). Interestingly, among the HCCs with high nuclear Tak1 expression, tumors with overexpression of glutamine synthetase, as an immunohistochemical surrogate parameter of WNT activation in, for example, CTNNB1-mutated HCC [33], were overrepresented (Table 1).

**Figure 4:**
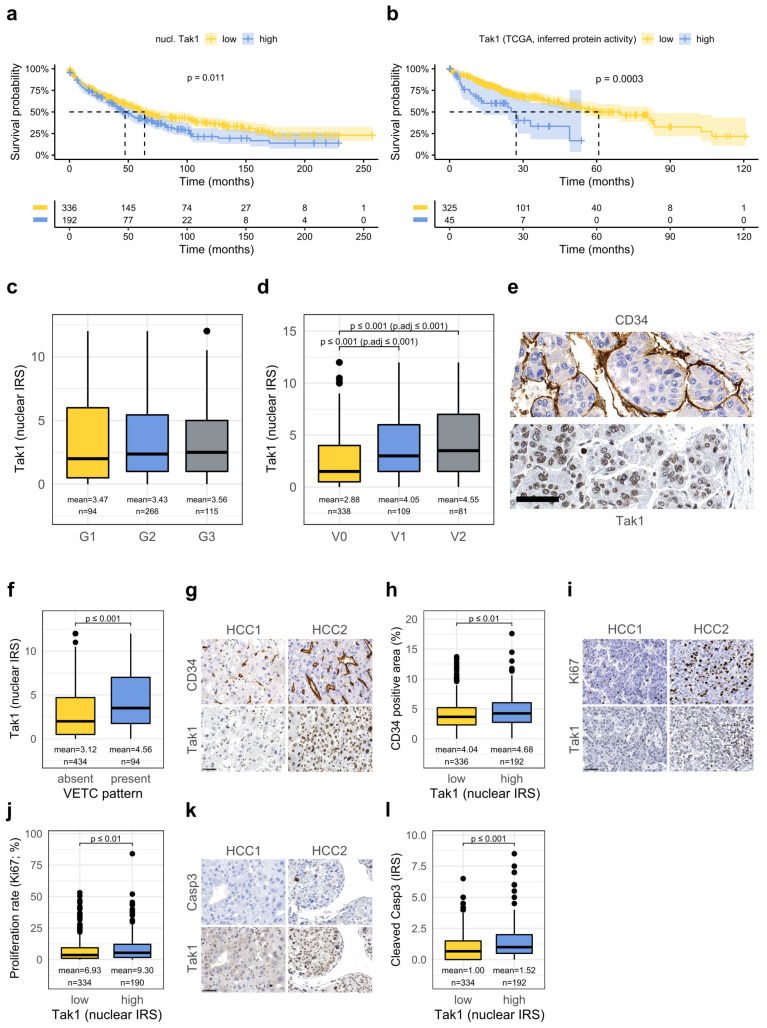
High Tak1 expression is associated with vascular invasion, vessels encapsulating tumor cell clusters, and unfavorable prognosis. (**a**) Kaplan–Meyer plot displaying overall survival with respect to high and low nuclear Tak1 protein expression as detected by immunohistochemistry (HR 1.34, 95% confidence interval 1.07–1.68, *p* = 0.011.) (**b**) Kaplan–Meyer plot displaying overall survival with respect to high and low estimated protein activity of Tak1 in the TCGA cohort (HR 2.31, 95% confidence interval 1.45–3.69, *p* ≤ 0.001). (**c**,**d**) Quantification of nuclear Tak1 immunoreactivity in human HCCs with respect to tumor grade (**c**) or vascular invasion (**d**). (**e**) Immunostaining of a HCC with VETC-pattern for CD34 (upper panel) and Tak1 (lower panel). (**f**) Quantification of nuclear Tak1 immunoreactivity in HCCs with respect to presence or absence of VETC-pattern. (**g**,**i**,**k**) Representative stainings for the indicated proteins with respect to high or low nuclear Tak1 staining. Scale bar: 50 µm. (**h**,**j**,**l**). Quantification of immunoreactivity of the indicated proteins with respect to high or low nuclear Tak1 staining.

High nuclear Tak1 immunoreactivity in primary HCCs (IRS > 3.58) was associated with HCCs of larger tumor size (Table 1) and reduced overall survival rates (Figure 4a). Multivariate analysis identified well-known prognostic factors, such as macrovascular invasion and Barcelona Clinic Liver Cancer (BCLC) stagewhereas high nuclear Tak1 expression was not among the statistically independent parameters (Table 2).

In confirmation of the data gained from our cohort, high Tak1 mRNA expression also predicted reduced survival in the independent, publicly available Cancer Genome Atlas (TCGA) cohort [24] (Figure S3a). Since mRNA levels do not necessarily correlate with protein activity and since Tak1 is known to be regulated by ubiquitination and phosphorylation [5], we made use of the metaVIPER algorithm to infer the protein activity of Tak1 from RNA sequencing data of the TCGA cohort [25]. This algorithm allows assessing the activity of a protein by integrative analysis of its interactome on the basis of its transcriptional targets. Analysis of RNA sequencing data can thereby not only reveal changes on mRNA level but also translational and posttranslational effects. In line with our previous findings, a high estimated protein activity of Tak1 in HCCs of the TCGA cohort also predicted short overall survival (Figure 4b). Additionally, Tak1 mRNA levels correlated well with estimated protein activity (Figure S3b).

In our cohort, no significant difference according to tumor grade was observed (Figure 4c), but significantly higher nuclear Tak1 immunoreactivity in HCCs with micro- or macrovascular invasion (Figure 4d) and vice versa a higher percentage of vascular invasion in tumors with high Tak1 expression (Table 1).

The increased vascular invasion may be interpreted in line with the increased migratory activity we observed in vitro upon Tak1 overexpression and may explain decreased patient survival. Tumor thrombi themselves, however, did not show significantly higher Tak1 expression when compared to the respective primary HCCs (Figure S2f). Moreover, tumors with the morphological feature “vessels encapsulating tumor clusters” as identified by anti-CD34 immunohistochemistry, which has been previously demonstrated to be associated with vascular invasion and short patient survival [34], exhibited significantly higher Tak1 levels (Figure 4e,f). Additionally, HCCs with high Tak1-expression also showed higher vascular densities, when compared to tumors with low Tak1 expression (Figure 4g,h), possibly indicating increased tumor angiogenesis.

Recently, different histological subtypes of HCCs have been recognized with characteristic morphology and distinct prognosis [35]. Interestingly, nuclear Tak1 expression was significantly lower in HCCs of the steatohepatitic subtype, when compared to HCCs of macrotrabecular-massive subtype or HCCs with no specific subtype (Figure S2g). The significance of this finding, however, is not yet clear.

Another explanation for the increased aggressiveness of HCCs with high Tak1 expression may be an increased proliferation rate in these HCCs [36]. Indeed, we detected a slightly, but significantly increased proliferation rate in HCCs with high Tak1 expression (Figure 4i,j). As previously published, deletion experiments have revealed an anti-apoptotic function of Tak1 [4], therefore, we hypothesized that high Tak1 levels may render tumor cells more resistant to apoptosis. Yet, contrariwise, human HCCs with high Tak1 expression even showed higher levels of cleaved caspase 3 as a marker for apoptosis (Figure 4k,l).

### 3.5. Combined Analysis of the Cyld-Tak1 Pathway Defines Distinct Prognostic Groups

**Figure 5:**
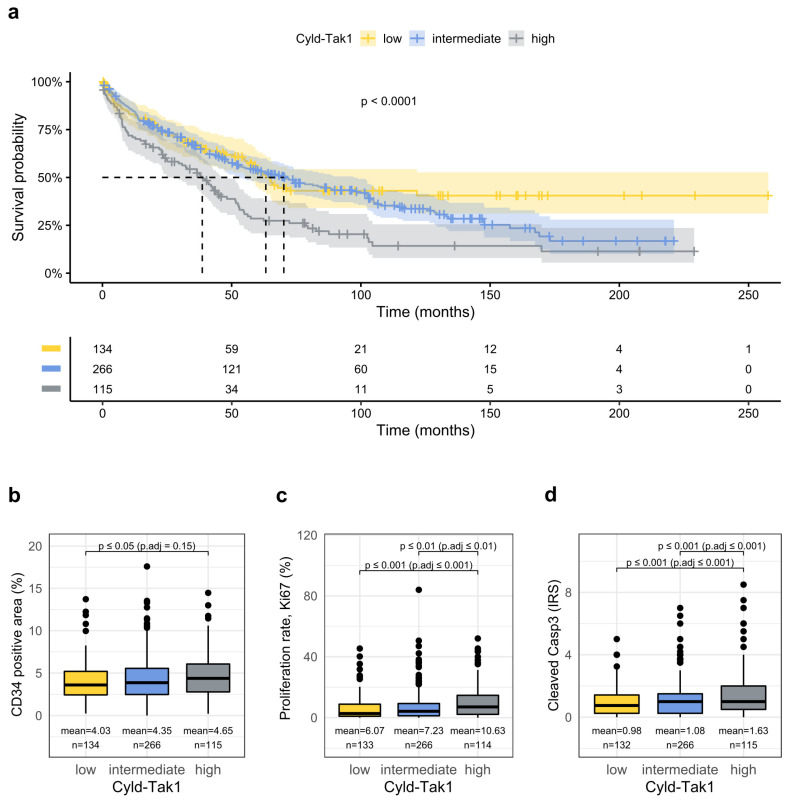
Combined analysis of the Cyld-Tak1 pathway defines distinct prognostic groups. (**a**) Kaplan–Meyer plot displaying overall survival with respect to high, intermediate, and low combined Cyld-Tak1 protein expression as detected by immunohistochemistry (intermediate vs. low: HR 1.15, 95% confidence interval 0.86–1.54, *p* = 0.4., high vs. low: HR 1.89, 95% confidence interval 1.31–2.72 *p* < 0.001). (**b**–**d**) Quantification of the indicated proteins with respect to high, intermediate, and low combined Cyld-Tak1 protein expression as detected by immunohistochemistry.

As the function of the deubiquitinase, Cyld as a negative regulator of Tak1 in the liver is known from several studies [13,14], we aimed to combine the data gained on Cyld and Tak1 expression with respect to prognosis. Remarkably, patients with both high nuclear Tak1 expression and low nuclear Cyld levels in HCC tissue, probably resembling the group with the most potent activation of the Cyld-Tak1 pathway, had a very short overall survival (Figure 5a). On the other hand, HCCs with low nuclear Tak1 and high Cyld levels, indicating the lowest activity of the pathway, had the highest survival rates, whereas the intermediate group, with either low nuclear Tak1 and low Cyld expression or high nuclear Tak1 and high Cyld expression, displayed an intermediate phenotype. Interestingly, these findings were also paralleled by vessel density (Figure 5b), the proliferation rate (Figure 5c), cleaved caspase 3 staining (Figure 5d), the frequency of vascular invasion, the presence of the VETC-pattern, and large tumor size (Table 3). Multivariate analysis, including the combined Tak1-Cyld classes, illustrated that high nuclear Tak1 and low nuclear Cyld expression clearly represents a strong and statistically independent prognostic factor.

## 4. Discussion

In this study, we analyzed the expression of the MAPKKK Tak1 in a large cohort of patients and correlated Tak1 expression with abundant clinicopathological data and provided further cell culture and mouse data in this respect. Predominantly the isoform Tak1A was expressed in the liver and HCC and was mainly localized in the cell nucleus. Tak1 was upregulated in human and murine HCCs, further induced in distant metastases, and high nuclear Tak1 expression was associated with short overall survival and vascular invasion. As this study represents a retrospective analysis, with all the limitations and potential biases applying to this type of study design, we confirmed the association of high Tak1 expression with poor prognosis also in the independent, publicly available TCGA dataset. Since Tak1 is well-known to be regulated post translationally, we additionally made use of the metaVIPER algorithm to infer protein activity from RNA sequencing data of the TCGA cohort [25]. Indeed, estimated protein activity of Tak1 paralleled mRNA levels and high inferred protein activity was associated with short overall survival. Another limitation of our study is that the HCC cohort was only composed of surgically resected HCCs, and advanced HCCs undergoing treatment with palliative intent may therefore be underrepresented.

Other studies have found high Tak1 expression to be associated with poor prognosis in thyroid [7], breast [9], and ovarian cancer [37]. Interestingly, peritoneal metastases of ovarian cancers also showed induction of Tak1 expression when compared to the primary tumors [37]. These studies, however, did not discriminate isoform-specific effects. Our in vitro data demonstrated, that especially the isoform Tak1A but not to the same extent the full-length isoform, Tak1B, mediates increased tumor cell migration, which is in line with previously published data in other cell types [18]. Our data also fit well to a recent publication by Cheng et al., that demonstrated decreased migratory behavior of HCC cell lines upon siRNA-mediated, non-isoform specific targeting of all Tak1 isoforms [36]. According to our data, TAK1A is by far the most prevalent isoform in the Huh7 cell line, therefore, we anticipate that the effect on migratory behavior is mainly mediated by Tak1A. Mechanistically, increased migratory behavior may be one explanation for the increased aggressiveness of HCCs with high Tak1 expression. Additionally, Tak1 also seems to be involved in the regulation of cell division, as we found a correlation of Tak1 with the proliferation rate in human HCCs, yet our overexpression experiments did not reveal differences in proliferation in Huh7 cells. Cheng et al., however, also demonstrated that knockdown of Tak1 leads to decreased proliferation in HCC cell lines and xenografted tumors [36], which indicates that there may be a certain level of constitutive Tak1 activity, possibly mediated by the constitutively active isoform Tak1A, that is driving tumor cell proliferation. As Tak1A shows the identical RNA sequence as full-length Tak1, with the exception of the absence of the 81 base pairs-long exon 12, it is, however, not possible to specifically target Tak1A without also affecting the full-length isoform Tak1B by, e.g., siRNA.

Although Tak1 is generally thought of as a cytoplasmic protein, we consistently found a predominantly nuclear immunoreactivity in human and murine liver and HCC tissue. This is in line with different studies that have also found nuclear localization in different cell types [38,39,40]. Additionally, Tak1 has also been demonstrated to shuttle to the nucleus after neddylation, a ubiquitin-like modification by SENP8 [41], and after stimulation with TGFβ [39] and WNT-1 [40]. The function of the shuttling of Tak1 to the nucleus is currently not clear. Interestingly, it has been demonstrated that there is increased neddylation and increased TGFβ-signaling in different chronic liver diseases and HCC [42,43]. Further investigations are needed to elucidate the functional role of nuclear Tak1 and whether the process of neddylation or the TGFβ- or WNT-signaling pathways play a role in its regulation.

Furthermore, our data from mouse experiments showed that Tak1 was also activated in the DEN mouse model of HCC. DEN is well-known to exert genotoxic effects [44] and also induced nuclear Tak1 expression in the non-tumorous liver tissue when compared to non-treated controls. Additionally, there was a further induction of Tak1 in HCCs that had developed in DEN-treated animals compared to the surrounding liver. As Tak1 has been implied in the DNA damage response [30], increased nuclear Tak1 expression upon DEN treatment may reflect an activation of this pathway. A possible downstream target of Tak1 in this respect may be IKKα(p45), a nuclear active form of IKKα, as it has been demonstrated to be activated by the BRAF-Tak1-p38 MAPK axis in response to DNA damage [45]. As there are several previously published studies that have described an increased activation of Tak1 in NASH, we also investigated the effects of high-fat, high-carbohydrate feeding on DEN-induced hepatocarcinogenesis. Although we only observed a trend towards an increase in nuclear Tak1 levels upon HFD, it seems plausible that increased activation of Tak1 may favor tumor growth and metastasis, as fatty acids have also been demonstrated to activate Tak1 and to drive invasion and metastasis [46]. In this context, we also made the interesting finding that Tak1 expression in HCCs correlated well with expression in the surrounding liver, both in human and mouse tissue. A persistent presence of inflammatory stimuli, possibly a combination of TNF, IL1, TGFβ, high levels of fatty acids, or LPS released from gut microbiota may lead to the activation of Tak1 both in the surrounding liver and HCC and may drive tumor progression [47,48,49].

Additionally, we confirmed previously published data, showing that Cyld was downregulated in human HCCs and that low nuclear Cyld levels were associated with short overall survival [32]. As there are robust previously published data that indicate that Tak1 is deactivated by deubiquitination by Cyld in mouse hepatocytes [14,19], we combined expression analyses of Tak1 and Cyld with respect to prognosis, which defined different groups with distinct prognosis. Patients with high nuclear Tak1 and low Cyld expression in HCC tissue showed significantly shorter overall survival, whereas vice versa, low nuclear Tak1 and high Cyld expression predicted longer survival times. In our opinion, a good explanation for this additive effect on prognosis is that this combined score better reflects the overall activity of the Cyld-Tak1 axis and that higher activity is correlated with worse prognosis. However, as Cyld is not specifically targeting solely Tak1 [50], we cannot exclude that this additive effect on prognosis is functionally related to effects on other targets. Interestingly, liver parenchymal-specific Cyld-deficient mice, which are known to show robust activation of Tak1 [19], did not show differences in nuclear Tak1 levels when compared to control animals, according to our data. Additionally, nuclear Tak1 did not correlate with nuclear Cyld expression in our large patient cohort. Nuclear translocation of Tak1, as discussed above, may therefore represent an additional mechanism in the complex regulation of Tak1 activity.

From a clinical point of view, Tak1 may represent a potential therapeutic target in the therapy of HCC. Tak1 inhibition has been considered a therapeutic option in colon, lung, and pancreatic cancers, entities that frequently exhibit KRAS mutations, which is based on the finding from preclinical models that Tak1 is exerting the prosurvival effects downstream of the rat sarcoma virus (RAS)/rapidly accelerated fibrosarcoma (RAF) signaling pathway [5]. In addition, in HCCs, an activation of the RAS-RAF-MEKK-ERK axis has been demonstrated, and the TKI sorafenib, which is approved for the treatment of unresectable HCC, is known to inhibit the RAF kinases [51,52]. Furthermore, HCCs with mutations in the BRAF gene have been reported to display a more aggressive clinical course with multiple tumor nodules and higher proliferation rates [53]. However, the efficacy of TKIs is limited by the development of acquired drug resistance with several different underlying mechanisms [3]. Further insight into the molecular mechanisms of TKI resistance are highly clinically relevant. In a preclinical study, inhibition of the BRAF-Tak1-p38 MAPK-IKKα axis has been demonstrated to sensitize tumor cells to different chemotherapeutic agents [45]. Very recently, a study demonstrated that Tak1 is upregulated in sorafenib-resistant HCC cell lines and that the inhibition of Tak1 is able to resensitize sorafenib-resistant cells to sorafenib-induced apoptosis both in vitro and in xenograft models [54]. Taken together, the inhibition of Tak1, especially in combination with other therapies, may represent an interesting potential molecular target in the therapy of HCC.

## 5. Conclusions

We here demonstrated in a large patient cohort, that Tak1 is activated in HCC independent of its etiology, further upregulated in distant metastases, and that activation of the Cyld-Tak1 pathway is associated with short patient survival, increased proliferation and vascular invasion. The commonly used DEN mouse model for HCC development also showed an activation of Tak1 in HCC. Interestingly, Tak1 is predominantly localized in the nucleus in liver and HCC tissue and is mainly present in form of its splice variant Tak1A. When Tak1A was overexpressed in Huh7 cells, we observed increased tumor cell migration, whereas overexpression of full-length Tak1 had no significant effect. Our data illustrate that the Cyld-Tak1 pathway plays an important role in hepatocarcinogenesis. Future studies on Tak1 should address isoform-specific effects and also the subcellular localization of Tak1, as well as crosstalk to the RAS-RAF signaling pathway.

## Figures and Tables

**Table 1:** Clinicopathologic data associated with high and low immunoreactive scores of Tak1.

Characteristic	Nuclear Tak1 Low	Nuclear Tak1 High	*p*-Value ^2^	Cytoplasmic Tak1 Low	Cytoplasmic Tak1 High	*p*-Value ^2^
	*n* = 336 (64%) ^1^	*n* = 192 (36%) ^1^		*n* = 455 (86%) ^1^	*n* = 73 (14%) ^1^	
Median age in years	62.6 (55.7, 69.9)	66.3 (59.2, 73.2)	<0.001	65.2 (56.6, 71.4)	60.4 (55.5, 66.7)	0.007
Max. diameter [mm]	36 (24, 68)	50 (30, 98)	<0.001	41 (25, 80)	38 (26, 92)	>0.9
Gender			0.12			0.1
Male	257 (76%)	158 (82%)		363 (80%)	52 (71%)	
Female	79 (24%)	34 (18%)		92 (20%)	21 (29%)	
Alcohol abuse	99 (29%)	56 (29%)	>0.9	134 (29%)	21 (29%)	>0.9
HCV	78 (23%)	28 (15%)	0.017	92 (20%)	14 (19%)	0.8
HBV	67 (20%)	31 (16%)	0.3	84 (18%)	14 (19%)	0.9
NASH	27 (8.0%)	15 (7.8%)	>0.9	34 (7.5%)	8 (11%)	0.3
Hemochromatosis	20 (6.0%)	4 (2.1%)	0.04	23 (5.1%)	1 (1.4%)	0.2
BCLC			<0.001			0.8
A	74 (22%)	20 (10%)		79 (17%)	15 (21%)	
B	201 (60%)	138 (72%)		292 (64%)	47 (64%)	
C	34 (10%)	27 (14%)		53 (12%)	8 (11%)	
D	27 (8.0%)	7 (3.6%)		31 (6.8%)	3 (4.1%)	
ECOG PST			0.7			>0.9
0–1	320 (96%)	185 (97%)		436 (96%)	69 (97%)	
2	7 (2.1%)	2 (1.1%)		8 (1.8%)	1 (1.4%)	
3	3 (0.9%)	2 (1.1%)		5 (1.1%)	0 (0%)	
4	5 (1.5%)	1 (0.5%)		5 (1.1%)	1 (1.4%)	
Liver cirrhosis			<0.001			0.5
Absent	102 (30%)	91 (47%)		164 (36%)	29 (40%)	
Present	234 (70%)	101 (53%)		291 (64%)	44 (60%)	
Child–Pugh Score			0.4			0.3
A	130 (52%)	58 (53%)		167 (53%)	21 (45%)	
B	94 (37%)	44 (40%)		115 (37%)	23 (49%)	
C	27 (11%)	7 (6.4%)		31 (9.9%)	3 (6.4%)	
Portal vein thrombosis (PVT)	34 (10%)	30 (16%)	0.062	56 (12%)	8 (11%)	0.7
Vascular invasion			<0.001			0.3
Absent	237 (71%)	101 (53%)		294 (65%)	44 (60%)	
Micro	58 (17%)	51 (27%)		89 (20%)	20 (27%)	
Macro	41 (12%)	40 (21%)		72 (16%)	9 (12%)	
Tumor grading			>0.9			0.008
G1	59 (20%)	35 (20%)		83 (20%)	11 (17%)	
G2	166 (56%)	100 (56%)		237 (58%)	29 (44%)	
G3	72 (24%)	43 (24%)		89 (22%)	26 (39%)	
MTM subtype			0.6			<0.001
no	305 (92%)	174 (93%)		421 (94%)	58 (79%)	
yes	28 (8.4%)	13 (7.0%)		26 (5.8%)	15 (21%)	
VETC			0.011			0.1
negative	287 (85%)	147 (77%)		369 (81%)	65 (89%)	
positive	49 (15%)	45 (23%)		86 (19%)	8 (11%)	
GS overexpression			<0.001			<0.001
no	286 (86%)	134 (70%)		352 (77%)	68 (96%)	
yes	48 (14%)	58 (30%)		103 (23%)	3 (4.2%)	

^1^ Median (IQR); *n* (%); ^2^ Wilcoxon rank-sum test; Pearson’s Chi-squared test; Fisher’s exact test.

**Table 2:** Univariate and multivariate Cox regression analyses of prognostic factors including Tak1 (overall survival).

	Univariate			Multivariate		
**Clinical Features**	**HR**	**std. Error**	***p*** **-Value**	**HR**	**std. Error**	***p*** **-Value**
Age (≥60 vs. <60)	1.43	0.12	0.003	1.44	0.13	<0.01
Sex (male vs. female)	1.07	0.13	0.637			
Alcohol abuse (true vs. false)	0.87	0.12	0.250			
Hemochromatosis (tr. vs. f.)	0.70	0.27	0.186	0.66	0.28	0.140
HBV (pos. vs. neg.)	0.89	0.14	0.400			
HCV (pos. vs. neg.)	1.07	0.14	0.608			
BCLC (B-D vs. A)	1.83	0.16	<0.001	1.58	0.18	0.010
AFP Serum (> vs. ≤200 ng/mL)	2.00	0.13	<0.0001			
**Pathologic features**						
Grading (G3 vs. G1/G2)	1.50	0.13	0.003	1.29	0.14	0.075
VI (micro vs. none)	1.49	0.14	0.004	1.34	0.16	0.064
VI (macro vs. none)	3.20	0.15	<0.0001	2.94	0.16	<0.0001
Macrotrabecular Subtype	1.96	0.19	<0.001			
nuclear Tak1 high	1.34	0.12	0.011	1.07	0.13	0.587

**Table 3:** Univariate and multivariate Cox regression analyses of prognostic factors, including the Cyld-Tak1 classes (overall survival).

	Univariate			Multivariate		
**Clinical Features**	**HR**	**std. Error**	***p*** **-Value**	**HR**	**std. Error**	***p-*** **Value**
Age (≥60 vs. <60)	1.43	0.12	0.003	1.42	0.13	<0.01
Sex (male vs. female)	1.07	0.13	0.637			
Alcohol abuse (true vs. false)	0.87	0.12	0.250			
Hemochromatosis (tr. vs. f.)	0.70	0.27	0.186	0.69	0.28	0.183
HBV (pos. vs. neg.)	0.89	0.14	0.400			
HCV (pos. vs. neg.)	1.07	0.14	0.608			
BCLC (B-D vs. A)	1.83	0.16	<0.001	1.50	0.18	0.024
AFP Serum (> vs. ≤200 ng/mL)	2.00	0.13	<0.0001			
**Pathologic features**						
Grading (G3 vs. G1/G2)	1.50	0.13	0.003	1.30	0.14	0.073
VI (micro vs. none)	1.49	0.14	0.004	1.24	0.16	0.172
VI (macro vs. none)	3.20	0.15	<0.0001	2.74	0.16	<0.0001
Macrotrabecular Subtype	1.96	0.19	<0.001			
intermed. comb. score (vs. low)	1.15	0.15	0.351	1.09	0.16	0.607
high comb. score (vs. low)	1.93	0.17	<0.0001	1.50	0.18	0.028

## Data Availability

No new data were created or analyzed in this study. Data sharing is not applicable to this article.

## Supplementary Materials

The following are available online at https://www.mdpi.com/article/10.3390/cancers14020430/s1, Figure S1: Additional data on mouse models and on Tak1 expression in HCCs of different etiology. Figure S2: Additional data on Tak1 expression in different etiologies. Figure S3: Additional data on Tak1 expression in the TCGA cohort. Figure S4: Uncropped WB original images. Table S1: List of antibodies used for immunohistochemistry. Table S2: Crude nutrients of experimental diets (both ssniff Spezialdiäten GmbH, Soest, Germany).
