# Supplementary material for: Transforming Growth Factor-β Activated Kinase 1 (Tak1) Is Activated in Hepatocellular Carcinoma, Mediates Tumor Progression, and Predicts Unfavorable Outcome
Source: Cancers (Basel). 2022 Jan 15;14(2):430. doi: 10.3390/cancers14020430 (PMC8774263; doi:10.3390/cancers14020430)
Supplement: Supplementary file 1 [file cancers-14-00430-s001.zip › cancers-1558872-Figures S1-S4.pdf]

cancers

MDPI

Supplementary materials: Transforming growth factor-β activated kinase 1 (Tak1) is activated in hepatocellular carcinoma, mediates tumor progression and predicts unfavorable outcome

Dirk Andreas Ridder, Lana Louisa Urbansky, Hagen Roland Witzel, Mario Schindeldecker, Arndt Weinmann, Kristina Berndt, Tiemo Sven Gerber, Bruno Christian Köhler, Federico Nichetti, Nadine Gehrke, Jörn Schattenberg, Stefan Heinrich, Wilfried Roth, and Beate Katharina Straub

Figure S1

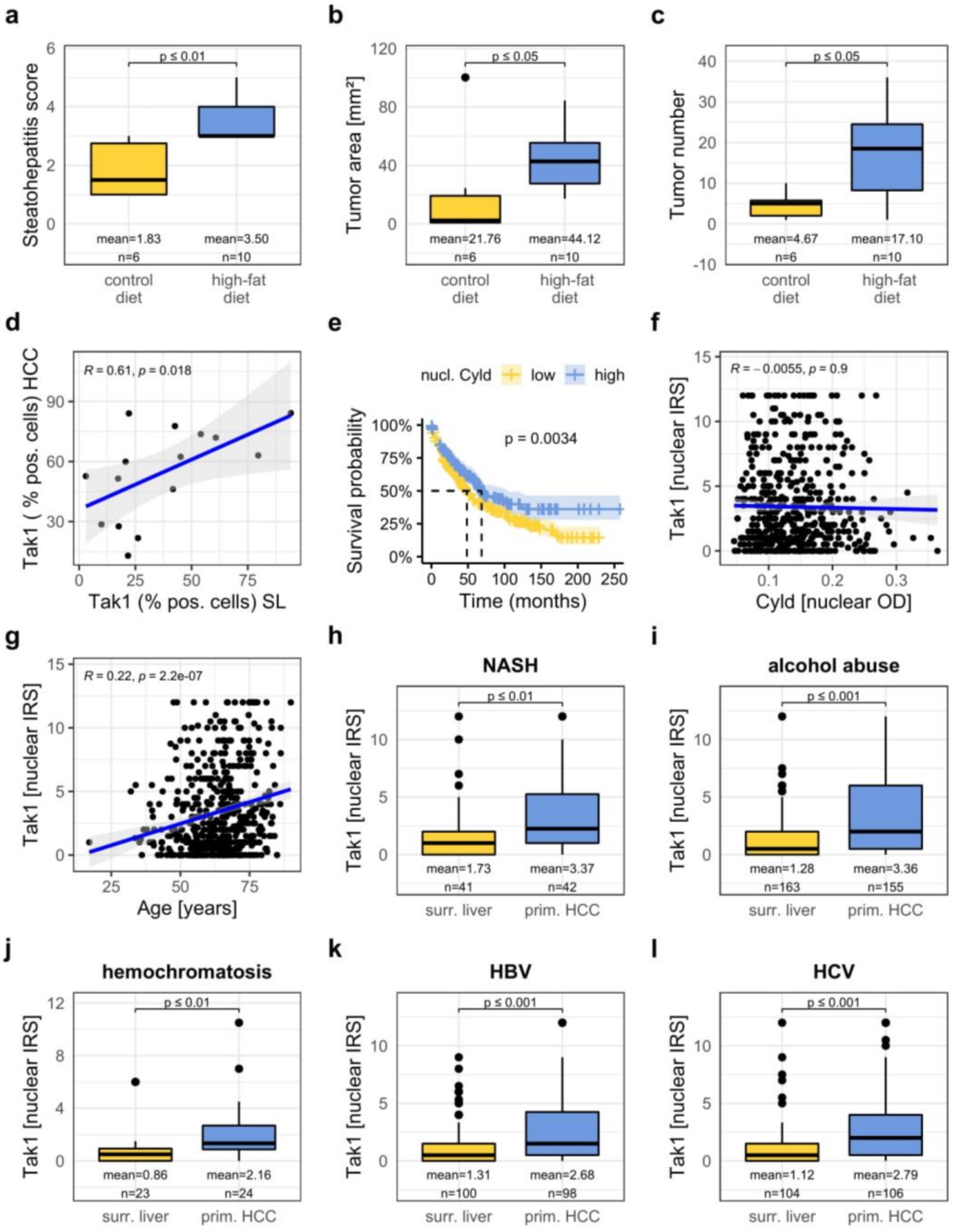

Figure S1. Additional data on mouse models and on Tak1 expression in HCCs of different etiology. (a) Steatohepatitis scores in livers of mice subjected to DEN-induced hepatocarcinogenesis with respect to control and high-fat, high-carbohydrate diet. (b) Quantification of histologically determined tumor size in mice subjected to DEN-induced HCC development with respect to control and high-fat, high-carbohydrate diet. (c) Tumor number in mice subjected to DEN-induced HCC development with respect to control and high-fat, high-carbohydrate diet by microscopic examination of the left lateral hepatic lobe. (d) Scatter plot of nuclear Tak1 immunoreactivity in DEN-treated mice on control or high-fat, high-carbohydrate diet in surrounding liver (x-axis) and HCC tissue (y-axis) (n = 15). (e) Kaplan–Meyer plot displaying overall survival with respect to high and low nuclear Cyld protein expression as detected by immunohistochemistry (HR 0.70, 95% confidence interval 0.55-0.89, p = 0.004.) (low: n = 309, high: n = 209). (f) Scatter plot of nuclear Cyld expression (x-axis) and nuclear Tak1 immunreactivity (y-axis) in human liver tissue (n = 515). (g) Scatter plot of age (x-axis) and nuclear Tak1 expression in HCC tissue (y-axis) (n = 528). (h) Quantification of nuclear Tak1 immunoreactivity in HCC and surrounding liver tissue as detected by immunohistochemistry in the subgroup of patients suffering from a NASH-induced HCC. (I) Quantification of nuclear Tak1 immunoreactivity in HCC and surrounding liver tissue as detected by immunohistochemistry in the subgroup of patients suffering from an alcoholic steatohepatitis-induced HCC.

Figure S2

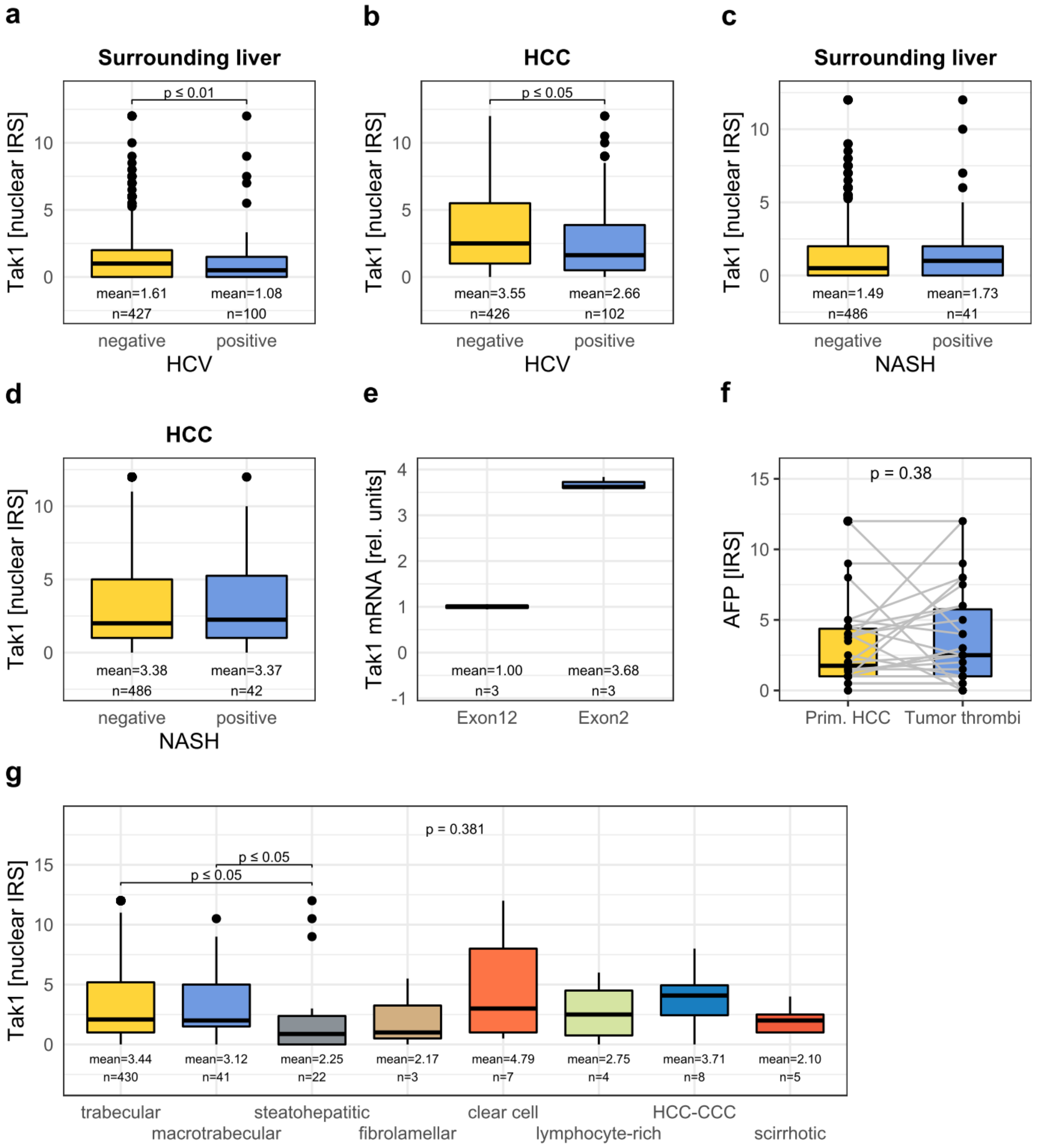

Figure S2. Additional data on Tak1 expression in different etiologies. (a) Quantification of Tak1 nuclear immunoreactivity as detected by immunohistochemistry in human liver tissue with respect to presence or absence of infection with HCV. (b) Quantification of Tak1 nuclear immunoreactivity in human HCCs that had developed upon HCV infection vs. other etiologies. (c) Quantification of Tak1 nuclear immunoreactivity in human liver tissue with respect to presence or absence of NASH. (d) Quantification of Tak1 nuclear immunoreactivity in human HCCs that had developed upon NASH vs. other etiologies. (e) Real-time RT-PCR from extracts isolated from Huh7 cells with primers specific for the constant exon 2 and the variable exon 12 of Tak1, indicating a predominance of the isoform Tak1A lacking exon 12. (f) Quantification of nuclear Tak1 immunoreactivity in tumor thrombi and the respective primary HCCs (n = 30). (g) Quantification of Tak1 expression according to morphological tumor subtype.

Figure S3

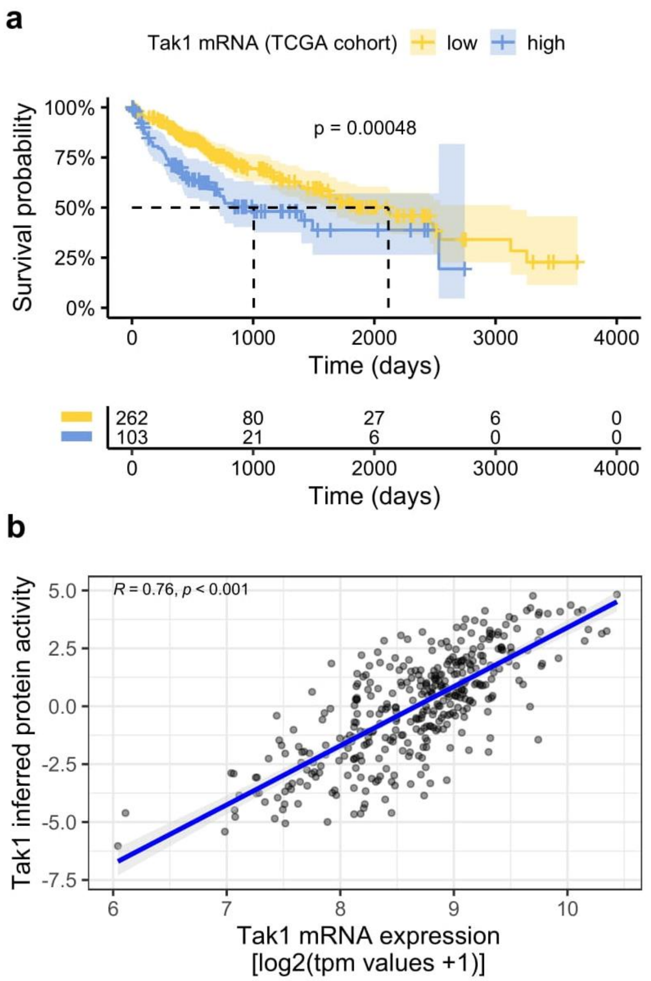

Figure S3. Additional data on Tak1 expression in the TCGA cohort.(a) Kaplan–Meyer plot displaying overall survival with respect to high and low expression of Tak1 mRNA in the TCGA cohort (HR 1.89, 95% confidence interval 1.31–2.72, p ≤0.001). (b) Scatter plot of Tak1 mRNA expression (x-axis) and estimated protein activity of Tak1 (y-axis) in human HCCs of the TCGA cohort (n = 371).

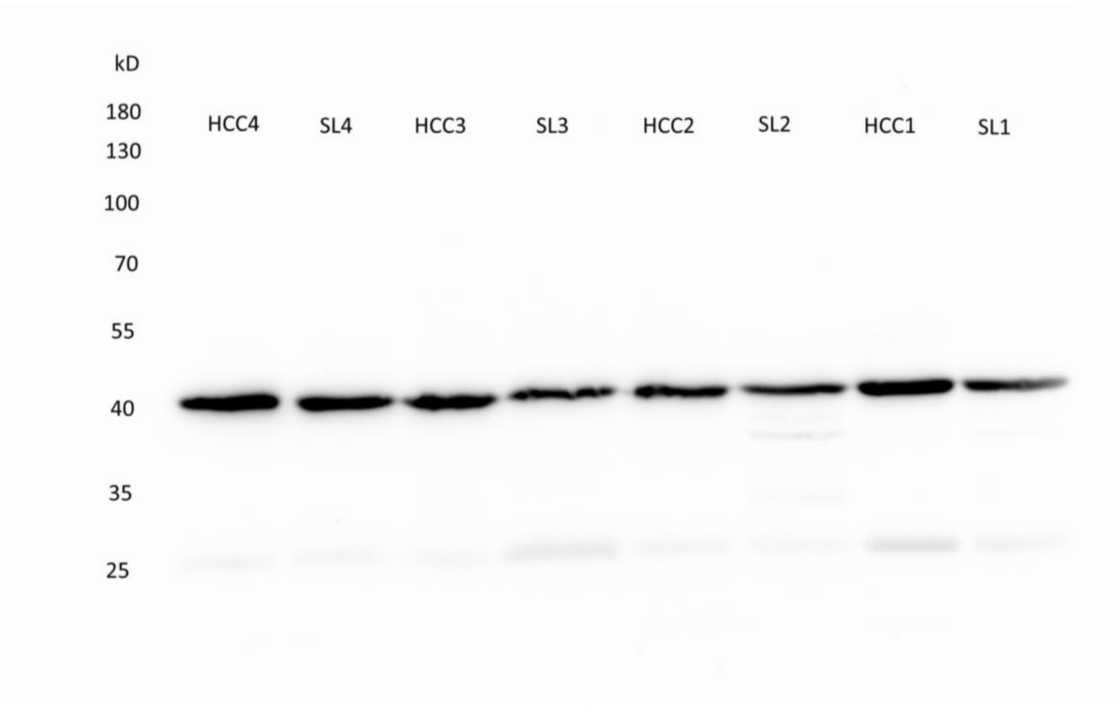

Original Blot to Figure 1c-Actin

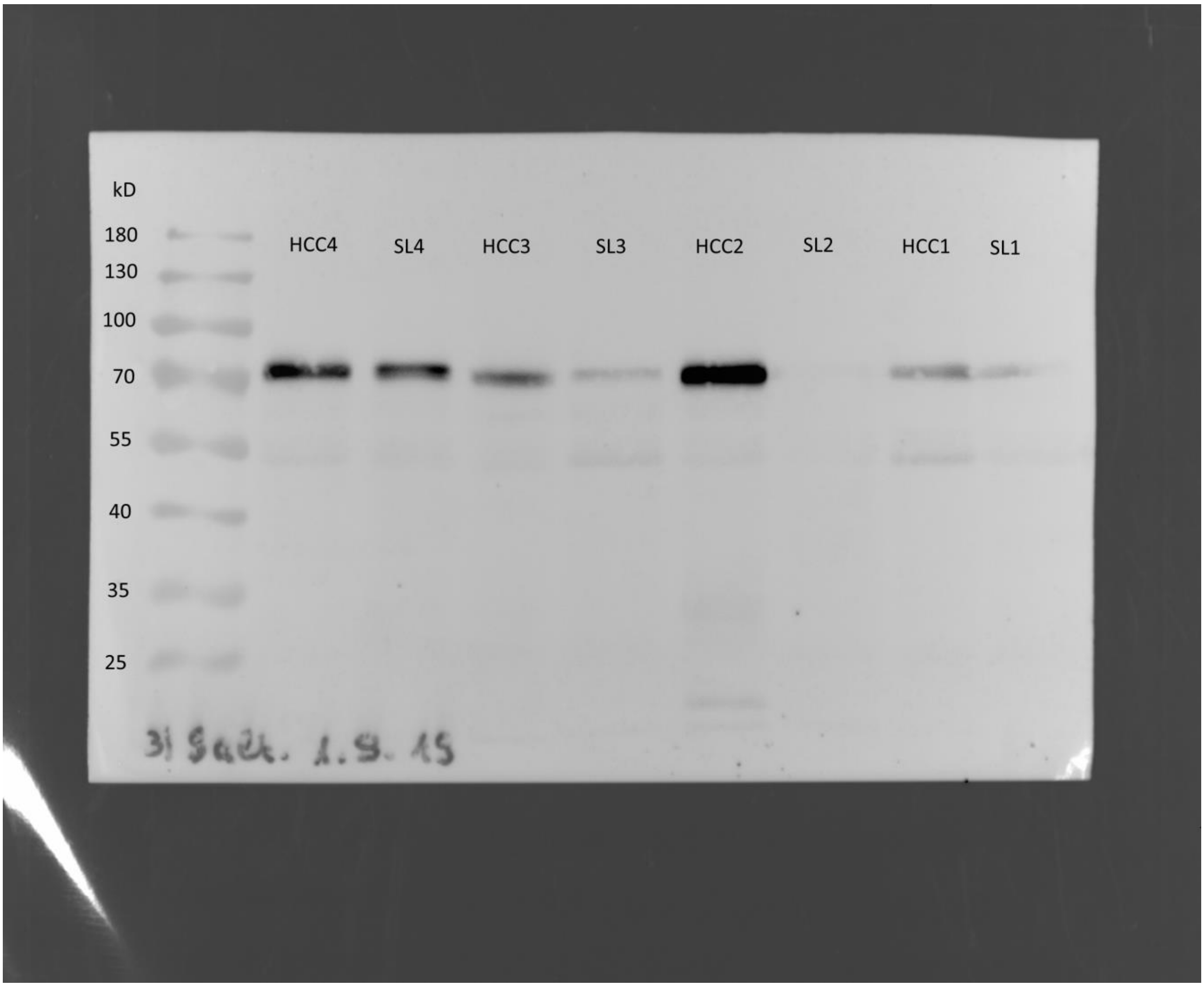

Original Blot to Figure 1c-Tak1

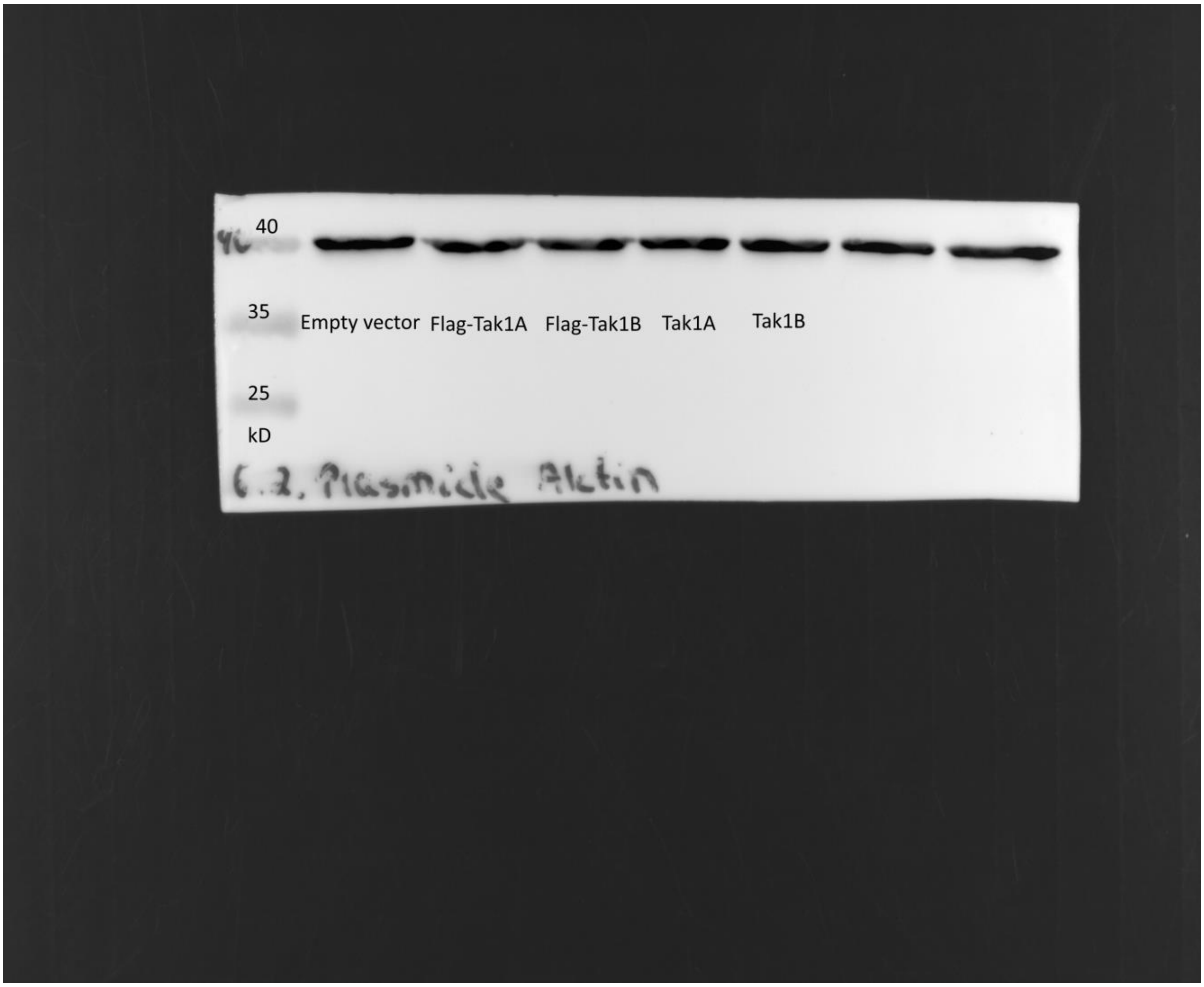

Original Blot to Figure 3a –Actin

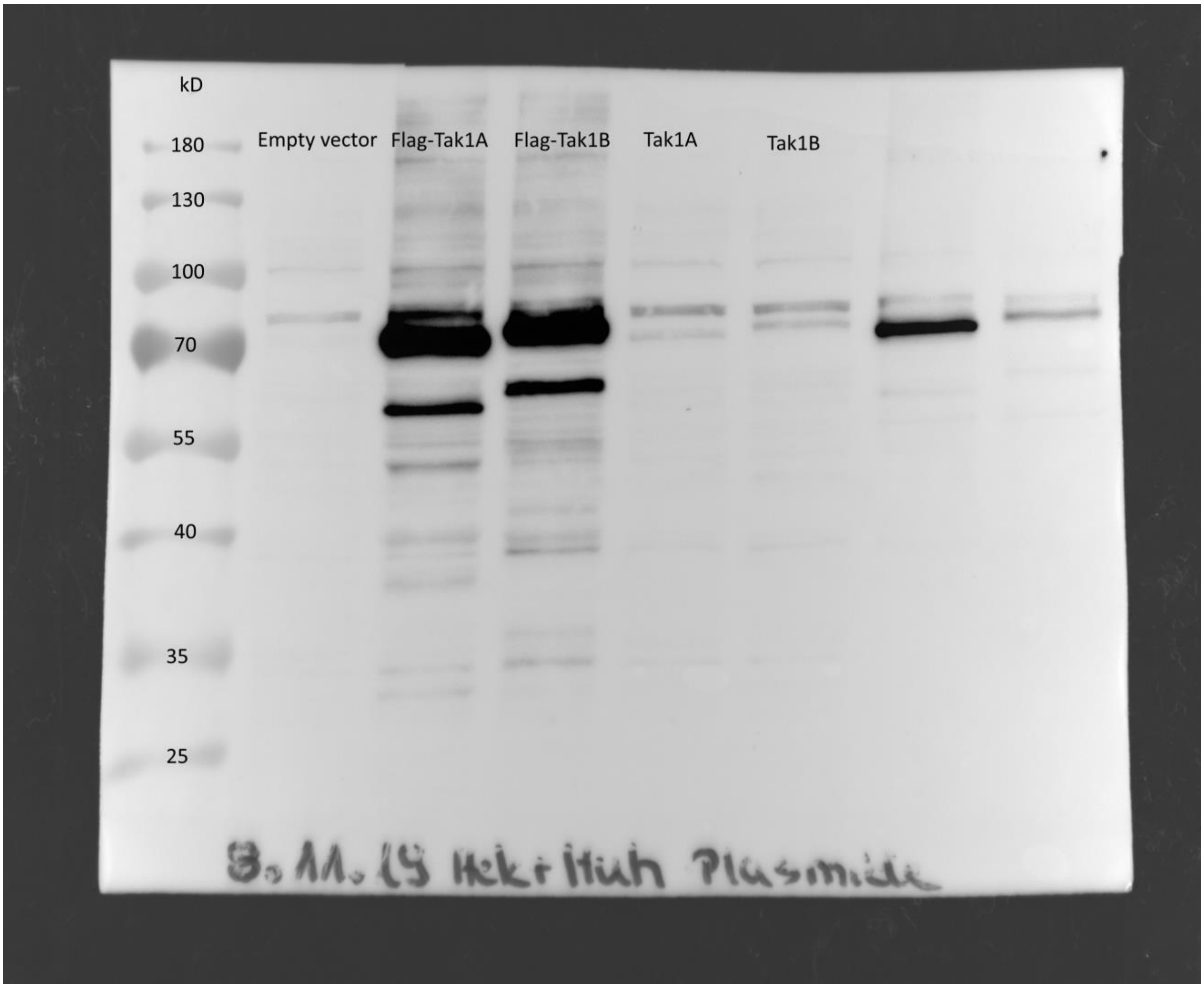

Original Blot to Figure 3a–Flag

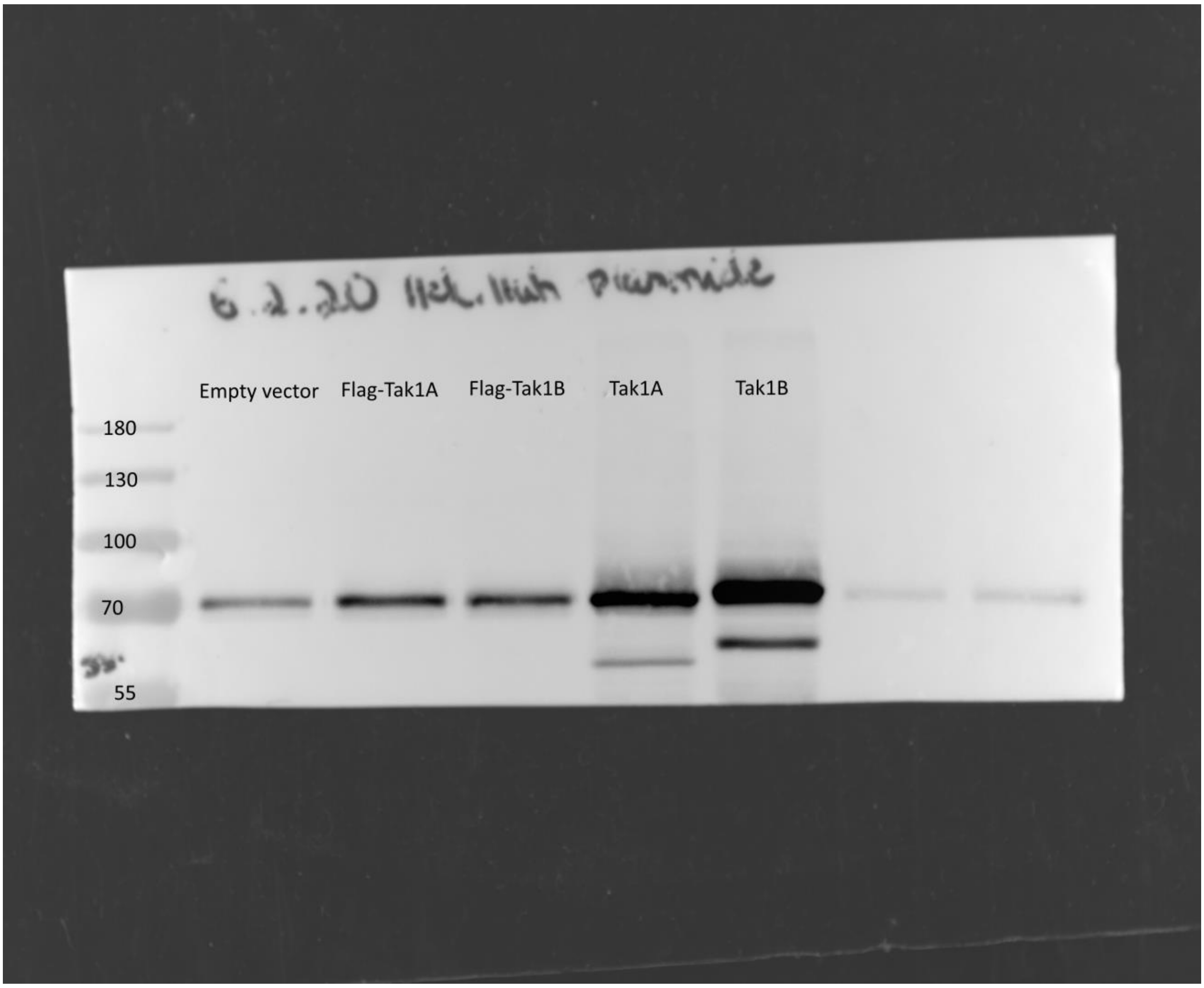

Original Blot to Figure 3a-Tak1.

Figure S4. Uncropped WB original images.
