# Supplementary material for: Transforming Growth Factor-β Activated Kinase 1 (Tak1) Is Activated in Hepatocellular Carcinoma, Mediates Tumor Progression, and Predicts Unfavorable Outcome
Source: Cancers (Basel). 2022 Jan 15;14(2):430. doi: 10.3390/cancers14020430 (PMC8774263; doi:10.3390/cancers14020430)
Supplement: Supplementary file 1 [file cancers-14-00430-s001.zip › cancers-1558872-Tables S1 and S2.pdf]

cancers MDPI

Supplementary materials

Transforming growth factor-β activated kinase 1 (Tak1) is activated in hepatocellular carcinoma, mediates tumor progression and predicts unfavorable outcome

Dirk Andreas Ridder, Lana Louisa Urbansky, Hagen Roland Witzel, Mario Schindeldecker, Arndt Weinmann, Kristina Berndt, Tiemo Sven Gerber, Bruno Christian Köhler, Federico Nichetti, Nadine Gehrke, Jörn Schattenberg, Stefan Heinrich, Wilfried Roth, and Beate Katharina Straub

Table S1. List of antibodies used for immunohistochemistry.

Antibody	Supplier	Cat. Nr.	Type	Dilution	Antigen Retrieval
Anti-cleaved Caspase-3	Cell Signal	9664	rabbit monoclonal	1:200	citrate buffer, pH 6.1
Anti-CD34	Dako	IR632	mouse monoclonal	ready to use	Tris/EDTA buffer, pH 9
Anti-Ki67	Dako	IR626	mouse monoclonal	ready to use	citrate buffer, pH 6.1
Anti-Tak1	Novusbio	JM73–19	rabbit monoclonal	1:25	citrate buffer, pH 6.1
Anti-Cyld	Abcam	ab137524	rabbit polyclonal	1:400	citrate buffer, pH 6.1
Anti-Glutaminsynthetase	Roche	760–4898	mouse monoclonal	ready to use	Roche, #950-124

Table S2. Crude nutrients of experimental diets (both ssniff Spezialdiäten GmbH, Soest, Germany).

Product No.		Surwit Diet (HFD) [HF/Sucrose] E15772-34	Control Diet (CD)[Soybean Oil] E15772-04
Casein	%	23.00	23.00
Corn stach, pre-gelatinized	%	-	27.00
Maltodextrin	%	15.30	15.30
Sucrose	%	17.00	17.00
Pur. Cellulose powder	%	1.80	5.20
DL-Methionine	%	0.10	0.10
L-Cystine	%	0.10	0.10
Mineral & trace element premix	%	5.50	5.50
Vitamin premix	%	1.00	1.00
Sodium phosphate, dibasic	%	0.30	0.30
Dye, red-yellow mix	%	0.10	-
Choline chloride	%	0.20	0.20
Coconut oil, hydrogenated	%	33.30	-
Soybean oil	%	2.30	5.30
ME, Atwater *	MJ/kg	22.80	15.70
Protein	kJ%	15	22
Carbohydrates	kJ%	26	65
Lipids	kJ%	58	13

* physiological fuel value.
